# Use of pre-hospital emergency medical services in urban and rural municipalities over a 10 year period: an observational study based on routinely collected dispatch data

**DOI:** 10.1186/s13049-019-0607-5

**Published:** 2019-04-02

**Authors:** Kathrin Hegenberg, Heiko Trentzsch, Stefan Gross, Stephan Prückner

**Affiliations:** Institut für Notfallmedizin und Medizinmanagement, Klinikum der Universität München, Ludwig-Maximilians-Universität München, Munich, Germany

**Keywords:** Pre-hospital emergency medical services, Emergency medical dispatch, Epidemiology, Health services use

## Abstract

**Background:**

Pre-hospital emergency medical services (EMS) are an integral part of emergency medical care. EMS planning can be achieved by analyzing patterns of use. However, long-term time trends of EMS use have rarely been studied. The objective of this retrospective study was to investigate utilization patterns over a ten year period, and to compare utilization trends between urban and rural municipalities and between events with and without prehospital emergency physician (PEP) dispatch.

**Methods:**

Routine data collected by 26 dispatch centers in the federal state of Bavaria, Germany, from 2007 to 2016 was analyzed. Emergency locations were classified into five levels of rurality. Negative binomial mixed effects regression models were fitted to predict emergency rates and to investigate differences in rates and utilization trends. Graphical representation methods were used to compare distribution of transport rates and distribution across daytime and weekday.

**Results:**

Twelve million two hundred thousand one hundred fifty-five dispatches assigned to 7,725,636 single emergencies were included. The mean number of emergencies per year and 1000 population (emergency rate) was 42.8 (±16.0) in rural municipalities and 80.7 (±9.3) in large cities. Compared to rural municipalities, cities had higher emergency rates without (IRR = 3.0, CI 2.2–4.0) and with pre-hospital physicians (IRR = 1.5, CI 1.2–2.0). Between 2007 and 2016, the absolute number of emergencies increased by 49.1%. Estimated annual percent change of emergency rates without physician activation ranged from 5.7% (CI 4.3–7.1) in cities to 7.8% (CI 7.6–7.9) in rural areas. Changes in emergency rates with physician attendance were lower, with estimated increases between 1.3 and 2.4%. The average proportion of patients transported to a hospital was lower in cities and remained unchanged. There were no considerable differences or changes in the distribution across daytime and weekdays.

**Conclusion:**

Differences between cities and other areas suggest that the planning of EMS should be targeted to regional characteristics. A substantial increase in emergency rates was observed across all areas of Bavaria, but did not impact transport rates or temporal distributions. Further research is needed to better understand the urgency of emergency events and reasons behind increasing EMS utilization.

## Introduction

Emergency medical services (EMS) are an integral part of emergency care and crucial for the provision of immediate medical care in the pre-hospital setting. To assure an appropriate response, proper planning of EMS infrastructure is paramount. An increasing year-on-year utilization of emergency ambulances over the past 20 years has been reported in many developed countries [1]. In order to provide an adequate number of mobile EMS units it is important to monitor the use of pre-hospital EMS and to respond to changing patterns. It is also important that adequate care is delivered in both, urban and rural regions.

Many factors influence the utilization of EMS. They include individual patient characteristic like age [2, 3], socioeconomic status and medical conditions [4, 5], patient preferences [6] and perceived priority [7] as well as system factors like the organization of primary care [8]. Urban-rural differences of ambulance use over time have not been investigated. Yet the use of health and emergency services and thus utilization trends likely differ between rural and urban regions, due to different patient preferences and healthcare infrastructure and different characteristics of patients.

The aim of this explorative study was therefore to describe the use of pre-hospital emergency medical services over a 10 year period and to compare urban and rural municipalities and emergencies with and without dispatch of emergency physicians. We investigate rates of pre-hospital EMS use, temporal distributions and transport rates.

## Methods

### Setting

The analyses in this retrospective observational study are based on ambulance dispatch data routinely collected between 2007 and 2016 in the German federal state of Bavaria. Any request for emergency medical assistance is made through the national emergency telephone number 112. For urgent but non-emergency conditions, on-call doctor services can be accessed through 116,117. 112 calls are managed by 26 regional dispatch centers which are run by different operators. Between 2007 and 2013, centers were gradually transformed to integrated centers which coordinate emergency and non-emergency ambulances as well as the fire brigade. Dispatchers are paramedics or firefighters who underwent additional dispatch training. EMS are organized as a two-tiered rescue system that consists of paramedic staffed ambulances and rapid response cars staffed with prehospital emergency physicians (PEP). Response decisions are made by the dispatcher who uses a non-standardized, keyword based dispatch protocol and a computer aided dispatch system. Emergencies that require PEPs are usually more complex and have a higher probability of unstable vital signs and/or for invasive interventions. PEP activation is triggered by one or more of the following criteria:

- loss of or severely impaired vital functions
- severe injuries, intoxication, massive blood loss, critical body temperature with suspected loss of vital functions
- fire, severe burns or scalding
- electrical or chemical accidents
- suspected danger to human life (e.g. shootings)
- psychiatric conditions that endanger the self or others
- accidents in water/ice
- imminent delivery

Activation of PEP can also be initiated at the discretion of the dispatch controller when a situation is unclear of for tactical reasons. This may be the case when transport times are expected to be long or when the response time would be long in case the PEP is not initially dispatched, but subsequently requested by the paramedics on site.

### Data source and sample

An electronic record is automatically created for each 112 call. 26 Bavarian dispatch centers transfer their EMS dispatch records to a central relational database on a monthly basis. The database contains a complete collection of every EMS dispatch record generated in Bavarian dispatch centers. Information about dispatches between the years 2007 and 2016 was extracted from the database if a dispatch met the following inclusion criteria: A dispatch had to be classified as a primary emergency that lead to the activation of a paramedic staffed ambulance, with or without support from a PEP. For those dispatches, regional location, time stamps and destination of the transport were extracted. If multiple dispatches were related to the same event, they were assigned to this event and analyzed as one single emergency. Multiple dispatches for the same event usually occur when an emergency physician is dispatched in addition to the paramedic staffed ambulance, or when more than one patient is involved or additional units are required for tactical support. Except for transport rates, all analyses in this paper refer to single emergency events, not corresponding dispatches.

Based on the location of the emergency, every emergency was assigned to one of the 2056 Bavarian municipalities. As geographic distances and infrastructure gradually vary between municipalities, we chose to compare five different levels of rurality. According to a classification by the Federal Institute for Research on Building, Urban Affairs and Spatial Development, each municipality was assigned to the respective level The assigned level depends on the size of the community and its regional importance. The regional importance of a community is stipulated in the regional development program and differentiates communities that can provide basic goods and services (e.g. doctor, pharmacy, bank branch, basic primary school, police station, train station), extended basic goods and services (e.g. secondary school, hospital, district authority) and specialized goods and services (e.g. specialized hospital, university, district court).

Levels are stratified as follows:

- Rural community: less than 5000 inhabitants, no provision of basic goods or services.
- Small town: minimum 5000 inhabitants and/or provision of basic goods and services.
- Large town: minimum 10,000 inhabitants and provision of basic goods and services.
- Medium-sized city: minimum 20,000 inhabitants and provision of extended basic goods and services.
- Large city: minimum 100,000 inhabitants and provision of specialized goods and services.

Data on the population of Bavaria was obtained from the Bavarian State Office for Statistics. 0.2% of emergencies occurred in areas that are not assigned to municipalities (e.g. forest areas). Since population figures are not available for these areas, dispatches to these areas were excluded from the analysis.

### Analysis

Emergency events are reported as absolute numbers and mean rates ± standard deviation (SD). To account for population growth, utilization is usually reported as annual rate per 1000 population (emergency rate). Analyses are usually stratified by municipality type and involvement of PEP (with PEP or without PEP).

A model was specified to assess the effect of year and municipality type on the emergency rate. It includes the number of emergencies in a municipality as the dependent variable and year and assigned municipality type as independent variables. To correct the number of events for population size, an offset variable was added to the model. Because of an over-dispersed count outcome variable, a negative binomial generalized linear model with logit link function was chosen [9] and fitted using the free R package lme4 [10]. To account for repeated measures on the same municipality over time [11], the model was extended to a mixed effects regression model with random intercept for each municipality. To assess whether the time trend in utilization was modified by municipality type, an interaction term between year and municipality type was included. We tested for statistical significance of the interaction effect by performing a likelihood ratio test. Separate analyses were run for emergencies with and without PEP. Regression coefficients from the fixed part of the model were exponentiated to obtain incidence rate ratios (IRR) and percent changes with 95% confidence intervals (CI).

Temporal distributions of emergencies and transport rates after dispatch were stratified by municipality type and PEP attendance. Transport was defined as an emergency event that led to dispatch of EMS units and that ultimately resulted in a transport of a patient to a hospital. Only vehicles equipped for patient transport were included in the analysis of transport rates. Boxplots, medians and interquartile ranges (IQR) describe the distribution of transport rates by municipality type, physician attendance and year. Statistical analysis was performed using R statistical software (R Foundation for Statistical Computing, Vienna, AT).

### Ethical aspects

The study was approved by the ethics committee of the medical faculty of the University of Munich (Project-No 17–813).

## Results

### Utilization and utilization trends

The total sample included 7,725,636 emergencies with 12,200,155 corresponding dispatches. Throughout the observed period, the overall minimum emergency rate at the municipality level was 1.7, the maximum rate was 330.6. Absolute numbers of emergencies and the mean emergency rate during the study period are shown in Table 1.

**Table 1:** Number of emergencies and mean emergency rate, 2007–2016

	overall		without PEP		with PEP	
	n	mean emergency rate (SD)	n	mean emergency rate (SD)	n	mean emergency rate (SD)
Total	7,725,636	46.6 (17.5)	3,993,450	20.0 (9.7)	3.732.186	26.6 (9.8)
Rural (*n* = 1371)	1,412,989	42.8 (16.0)	589,129	17.5 (8.0)	823,860	25.2 (9.4)
Small town (*n* = 458)	1,275,160	49.0 (17.1)	565,913	21.3 (9.5)	709,247	27.7 (9.8)
Large town (*n* = 161)	1,335,647	60.9 (13.2)	650,602	29.4 (9.8)	685,045	31.5 (8.9)
Medium-sized city (*n* = 58)	1,48,4817	73.1 (13.1)	769,047	37.3 (9.7)	715,770	35.9 (8.0)
Large city (*n* = 8)	2,217,023	80.7 (9.3)	1,418,759	44.4 (8.4)	798,264	36.4 (9.1)

PEP: pre-hospital emergency physician

IRRs are shown in Table 2. For both, emergencies with and without PEP, the estimated average emergency rate was statistically significantly higher the larger the assigned municipality type, using rural communities as a reference. Yet differences in estimated average emergency rates between municipalities were smaller when PEPs were dispatched: Compared to rural communities, the emergency rate without PEP was three times higher in large cities (CI 2.2–4.0, *p* < 0.001), whereas with PEP, the rate was 1.5 times higher (CI 1.2–2.0, *p* < 0.001).

**Table 2:** Incidence rate ratios for emergency rate, 2007–2016

	Without PEP	With PEP
	IRR (CI)	IRR (CI)
Rural (reference)	1	1
Small town	1.2 (1.2–1.3)*	1.1 (1.1–1.2)*
Large town	1.8 (1.6–1.9)*	1.3 (1.2–1.3)*
Medium-sized city	2.2 (2.0–2.5)*	1.5 (1.4–1.7)*
Large city	3.0 (2.2–4.0)*	1.5 (1.2–2.0)*

*p < 0.001

PEP: pre-hospital emergency physician

**Fig. 1:**
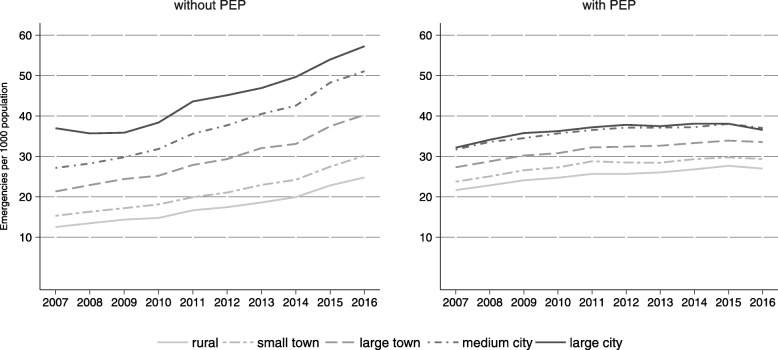
Time trends of mean emergency rate, 2007–2016

In a 10-years-period of time, there was a 49.1% absolute increase in emergencies and a 51.2% absolute increase in dispatches. While the average overall emergency rate in 2007 was 37.2 (± 15.2), it increased to 56.4 (± 22.9) in 2016 (+ 51.9%). The increase was smaller for emergencies with PEP (from 22.9 ± 9.6 to 28.3 ± 11.9) as compared to emergencies without PEP (from 14.3 ± 8.1 to 28.1 ± 13.6). Figure 1 shows time trends of emergency rates by municipality type and PEP attendance. The mean rate was higher the larger a municipality in all years, except for emergencies with PEP located in medium-sized and large cities, which show event rates comparable to each other. Mean rates of emergencies were more similar across small and large municipalities if PEPs were engaged into a rescue missions. An increase in rates between the years 2007 and 2016 was observed across all municipalities and for both, emergencies with and without PEP.

Estimated average annual percentage change is shown in Table 3.

**Table 3:** Estimated average annual percentage change of emergency rates with corresponding confidence intervals, 2007–2016

	Rural	Small town	Large town	Medium-sized city	Large City
Without PEP	7.8 (7.6–7.9)	7.8 (7.4–8.2)	7.5 (7.0–8.0)	7.8 (7.2–8.5)	5.7 (4.3–7.1)*
With PEP	2.4 (2.3–2.5)	2.2 (1.8–2.6)	2.4 (1.9–2.9)	1.7 (1.0–2.4)*	1.3 (− 0.2–2.8)

*p* value for interaction of year and municipality type (rural = reference), * *p* < 0.001

*PEP* pre-hospital emergency physician

In rural communities (reference), the increase by one year leads to an increase of the emergency rate by 7.8% (CI 7.6–7.9 *p* < 0.001). The interaction terms show that this effect was barely modified by municipality type, meaning that the increase was similar between municipalities of different size. Large cities were the exception. Only large cities experienced a statistically significant (*p* < 0.001) lower increase of 5.7% (CI 4.3–7.1). When PEPs were dispatched, the estimated annual increase in rural communities was 2.4% (CI 2.3–2.5, *p* < 0.001). A statistically significant difference in change of rates was only observed in medium-sized cities, where the estimated annual increase was only 1.7%. The increase in large cities was even lower. However, the confidence interval was large.

### Temporal distribution of emergencies

**Fig. 2:**
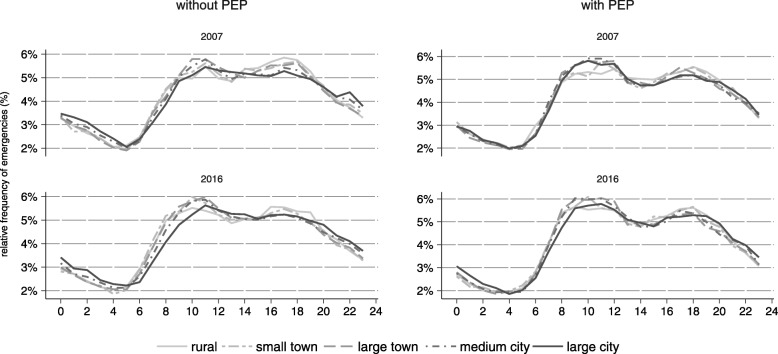
Distribution of emergencies across daytime, 2007 and 2016

Figure 2 shows that emergency rates picked up at 6 am and peaked around midday. Another peak was observed around 5 pm. Peaks were more pronounced in emergencies with PEP. Between 2007 and 2016, the distribution slightly shifted from nighttime to daytime, with a slightly smaller proportion of emergencies happening between 10 pm and 6 am in recent years (21.8 ± 5.6%) in 2007 vs 20.5 ± 5.1%) in 2016).

**Fig. 3:**
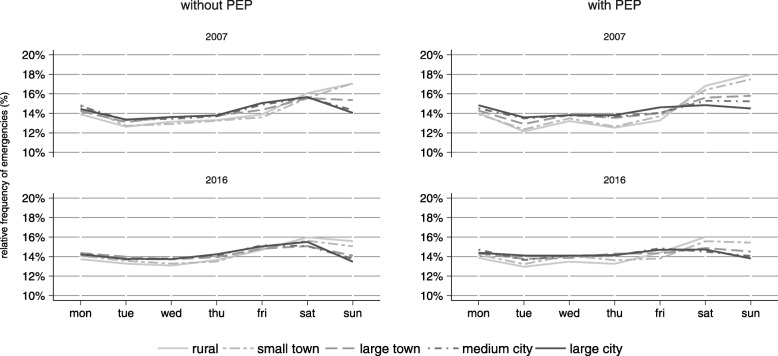
Distribution of emergencies across weekday, 2007 and 2016

Figure 3 shows the proportion of emergencies for each day of the week in 2007 and 2016. The comparison between municipalities of different size shows that, the smaller a municipality, the higher the mean proportion of emergencies at weekends (large cities: mean 29.2% (± 0.5); rural communities 33.0% (± 3.1)). Over the years, a small shift from weekends towards weekdays was observed: The overall mean proportion of emergencies at weekends fell from 33.6% (± 6.7) in 2007 to 31.3% (± 6.0) in 2016.

### Transport rates

**Fig. 4:**
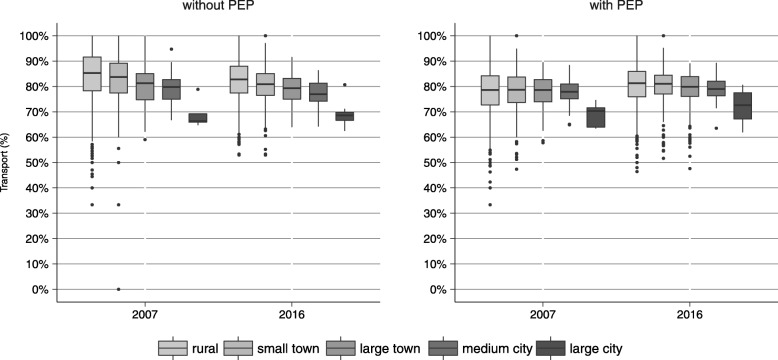
Distribution of transport rates, 2007 and 2016

Figure 4 shows boxplots of the proportion of dispatches to a municipality that actually led to the transport of a patient to a hospital for the years 2007 and 2016. Overall transport rates were higher for emergencies without PEP as compared to emergencies with PEP (with PEP: 80.0% (IQR: 9.8); without PEP: 82.8% (IQR: 11.5)). For emergencies without PEP, transport rates decreased with increasing size of a municipality. The median rate for rural municipalities in 2016 was 82.8% (IQR: 10.6), compared to a median transport rate of 68.6% (IQR: 3.1) in large cities. For missions with PEP, differences in transport rates with respect to rurality were less evident. However, with median transport rates of 71.6% in 2007 and 77.5% in 2016 transport rates in large cities were still lower than in all other areas. Compared to 2007, slightly lower median transport rates were observed when there was no additional physician dispatch (median of 84.1% in 2007 and 81.8% in 2016), whereas median transport rates for emergencies with PEP were slightly higher in 2016 compared to 2007 (median of 78.6% in 2007 and 81.0% in 2016).

## Discussion

Emergency rates differed between rural and urban regions, with higher rates in urban municipalities. Over a period of ten years, a substantial increase in the number of emergencies was observed. The increase of emergencies that did not require an emergency physician on scene was much higher than the increase in emergencies with emergency physician attendance. Time trends of utilization were similar between municipalities of different size, except for cities, where the yearly increase was smaller. Transport rates were similar between rural and urban regions when a physician was dispatched and higher in smaller municipalities when an emergency physician was not present, but did not change over time. Temporal patterns of pre-hospital utilization were similar between urban and rural regions and over time.

### Emergency rates and differences between urban and rural communities

The number of emergencies per 1000 population was higher the larger a municipality. Comparison with other studies is made difficult by the heterogeneous organization of pre-hospital EMS and definition of urban and rural regions. Few other studies report urban-rural differences in EMS use and find identical incidence of emergency transports to emergency departments in urban and rural areas [12] as well as a higher incidence of low-urgency emergencies in rural areas [13].

Coordinated planning of EMS structures in Bavaria has been initiated in 1999 to prevent both large gaps in provision of EMS and extreme clustering of EMS structures [14]. It is therefore unlikely that differing emergency rates are the result of an unbalanced distribution of pre-hospital EMS infrastructure. An obvious cause of higher emergency rates in larger municipalities is that daytime population density is higher in urban regions, mostly because of inbound commuters. Another cause would be a worse health status of urban citizens, which we could not control for in our study. Health status is associated with age and there is evidence that a large proportion of EMS use can be attributed to the elderly [3, 2]. According to official statistics the average proportion of people aged 75 years and older ranged between 8 and 10%. This difference however, does not seem big enough to fully explain varying rates. Health status is also associated with socioeconomic deprivation. Deprivation was associated with higher emergency call rates in England [15], and the observed association between population density and deprivation could explain higher rates cities. Another cause may be that people in larger municipalities have different preferences regarding emergency care alternatives. Connection to a general practitioner [16], a stronger sense of ‘relationship’ and more complex decision-making in emergency situations [17] distinguish rural from urban patients. Citizens of urban areas might more often choose to call for an ambulance, whereas rural citizens consider other options first, especially in situations that do not seem life threatening. Rural areas are dealing with a higher share of emergencies with PEP. This could reflect differences in decision making or disease spectrum, but could also be due to an adapted dispatch strategy in areas where times to get to the scene and transport times to hospital are long.

### Time trends of emergency rates and differences between urban and rural communities

With an increase of 49.1%, the absolute number of emergencies changed substantially during the 10 year period. The mean number of emergencies per 1000 population in Bavarian municipalities increased from 37 to 56. Thus the increase in emergencies was much higher than population growth. Rather uniform increases were observed across municipalities of different size, with the exception of large and medium-sized cities, where increases were lower. Large confidence intervals for large cities are probably due to the smaller sample of large cities. There are numerous possible explanations behind increasing utilization and the contribution of different factors is unclear. Differences might partly be due to differing age structures. Official population data show that, depending on the municipality type, the number of people aged 75 years or older increased between 28 and 36%, with urban areas facing the smallest increases. However, a backward projection with Bavarian dispatch data has already shown that only a small proportion of the total increase in EMS use between 2004 and 2011 can be attributed to demographic change [3]. A part of the total demand for emergency medical services in cities might be absorbed by alternative health services.

### Time trends of emergency rates and differences between emergencies with and without physician dispatch

The increase in emergency rates was higher for emergencies that did not require the additional dispatch of a physician, regardless of the size of a municipality. This could indicate changing needs, with a shift towards conditions that do not require PEP activation. It could also indicate that dispatchers are not able to match calls with an appropriate response. The supply and the adequate regional distribution of ambulances and PEPs are coordinated according to legal requirements. If a patient requires PEP treatment according to the dispatch catalogue, the nearest PEP will be dispatched. PEP shortage might therefore lead to longer response intervals, but is unlikely to put a cap on the rate of emergencies with physicians. Compared to criteria for PEP dispatch, criteria for dispatch of paramedic staffed ambulances are less clearly defined. Appropriateness of utilization of emergency services has been questioned by different authors in different countries [18–20], and an increase of non-specific diagnoses has been reported [21]. A part of increase in missions where the presence of a physician is not required could be attributed to the fact that dispatchers are not able to match unspecific and less urgent calls with a response other than a paramedic staffed ambulance. Dispatch for non-specific complaints and non-urgent diseases would be a problem for ambulance crews, as they seem to have difficulties in dealing with patients with non-serious clinical needs [22].

### Time trends of transport rates and differences between urban and rural regions and emergencies with and without physician dispatch

There are different reasons for non-transport. Some emergencies turn out to be a false alarm, some patients are already dead on arrival or refuse transport, or on-scene care was sufficient enough to decline the state of emergency. PEP are usually confronted with more serious conditions thus chances are higher that a patient is pronounced dead on scene and does not undergo transportation. PEP may also find it easier to decide if a patient needs transport to a hospital or can be left at home. Lower transport rates in urban areas, especially large cities, indicates that surrounding infrastructure might play a role, but could also be explained by a higher amount of “false alarms” and alarms for conditions involving patients refusing to be transported. There was no considerable change in transport rates over time.

### Time trends of temporal patterns and differences between urban and rural regions

Time of day patterns show a typical bimodal distribution with peaks in the morning and evening and less activity at night [23, 24], which was also present in our data and did not vary by the size of a municipality or year. A higher demand on Fridays [25] and weekends has been found by other authors, especially for alcohol-related and trauma cases [24]. We also observed a slightly higher proportion of emergencies on weekends. This was especially true for smaller municipalities, which are probably less affected by commuter flows to cities on weekends, or which are recreational areas and are therefore more crowded on weekends. In spite of the strong increase in the number of emergencies, patterns remained almost unchanged.

### Future perspectives

Further research is needed to better understand the urgency of emergency events and to identify non-emergency situations. This should be followed by improving triage mechanisms at dispatch and by defining multiple levels of response that best match patients’ needs. Difficult triage decisions and fear of legal implications might lead paramedic crews to always convey patients to a hospital. The development of protocols could help ambulance crews choose save alternatives to transport to hospital. New concepts should also take patients’ perspectives and preferences regarding the access to emergency care into account. To better predict future utilization and explain observed trends there is also a need for a more extensive, uniform and consistent data collection that includes patient-specific medical and sociodemographic data and information about access to healthcare infrastructure.

## Limitations

Data were routinely collected. As dispatch records are created automatically, the documentation of a dispatch is a reliable measure for the activation of an EMS unit and we believe that the number of registered emergencies is accurate. Yet there is some degree of uncertainty regarding the correctness and completeness of time stamps and the destination of transport due to data entry errors. We believe that bias from data entry errors and missing data is small, as the database holds every dispatch record generated in a Bavaria dispatch center and time stamps and transport destinations are important in the subsequent dispatch process and therefore usually well documented. Because of the lack of standardized dispatch algorithms, the choice of type of ambulance and frequency of additional PEP dispatch might vary between the 26 dispatch centers. We believe that potentially different dispatch strategies do not bias the results, as emergencies assigned to the same level of rurality were handled by many different centers. Our study fails to provide explanations for causes of trends. Many explanatory variables of interest were not included in our model, because they are not part of a consistent routine data collection. Insights in pre-hospital EMS utilization and urban rural differences from our study may be applicable for Bavaria only and might not be transferable to other regions with different population composition and healthcare infrastructure.

## Conclusion

A substantial increase in emergency rates in Bavaria was observed across all areas over the past 10 years. However, transport rates and temporal distributions remained unchanged. Reasons behind differing emergency rates in urban and rural communities and reasons behind increasing utilization remain unclear. However, EMS use differs between rural and urban areas and regional characteristics should be taken into account when planning pre-hospital emergency medical infrastructure.

## Abbreviations

CI: Confidence interval
EMS: Emergency medical services
PEP: Prehospital emergency physician
SD: Standard deviation
